# Phytofabricated Silver Nanoparticles From *Vaccinium arctostaphylos* L.: Anticancer Activity Against Breast Cancer Cells via Apoptosis Induction and Growth Suppression

**DOI:** 10.1002/fsn3.71202

**Published:** 2025-11-12

**Authors:** Nazila Soleimani, Zahra Keshtmand, Nader Goodarzi, Mohsen Akbaribazm

**Affiliations:** ^1^ Student Research Committee, Khoy University of Medical Sciences Khoy Iran; ^2^ Department of Biology, CT.C. Islamic Azad University Tehran Iran; ^3^ Department of Basic Sciences and Pathobiology, Faculty of Veterinary Medicine Razi University Kermanshah Iran; ^4^ Department of Basic Medical Sciences Khoy University of Medical Sciences Khoy Iran

**Keywords:** breast cancer, green synthesis, MCF‐7, silver nanoparticles, *Vaccinium arctostaphylos* L.

## Abstract

This study investigates the anticancer efficacy of phytofabricated silver nanoparticles (VACAgNPs) derived from *Vaccinium arctostaphylos* L. (VAC) against molecularly distinct breast cancer subtypes (MCF‐7: hormone‐responsive; 4T1/MDA‐MB‐231: triple‐negative). Hydroalcoholic VAC fruit extract, characterized by LC‐ESI/MS (71 phenolic compounds, including quercetin‐3,7‐diglucoside and kaempferol galloylglucoside) and GC–MS (methyl 3,5‐bis(ethylamino)benzoate, 26.71%), served as a dual reducing‐capping agent for green nanoparticle synthesis. FE‐SEM and DLS confirmed spherical VACAgNPs (mean size: 124.67 nm; zeta potential: −6.32 mV) with 66.82 wt% Ag content. In vitro assays revealed dose‐ and time‐dependent cytotoxicity, with 100 μM VACAgNPs reducing viability to 41.0% ± 6.1% (MCF‐7), 39.3% ± 6.1% (4T1), and 30.6% ± 5.6% (MDA‐MB‐231) at 48 h, outperforming cisplatin in caspase‐3‐deficient MCF‐7 cells. Mechanistic studies demonstrated ROS‐mediated apoptosis via Bax/Bcl‐2 ratio modulation (14.0‐fold increase in MDA‐MB‐231) and Caspase‐3 activation (3.91‐fold), alongside oxidative stress regulation: FRAP increased to 6.42 ± 0.81 μmol/mL (49.6% above control), while MDA and NO decreased by 59.2% and 31.5%, respectively. The nanoparticles' bioactivity correlated with VAC's antioxidant profile (TPC: 91.06 ± 9.16 mg GAE/g; FRAP: 9520.12 ± 56.46 mmol Fe^2+^/mg), overcoming phytochemical bioavailability limitations through enhanced targeting. These findings establish VACAgNPs as a sustainable therapeutic candidate, offering subtype‐agnostic efficacy via dual apoptosis induction and redox modulation, while mitigating chemotherapy‐associated toxicity. The study provides critical preclinical evidence for advancing plant‐nanoparticle synergism in precision oncology.

## Graphical abstract

This study demonstrates that phytofabricated silver nanoparticles (VACAgNPs) from *Vaccinium arctostaphylos* L. exhibit significant anticancer activity against breast cancer cells, inducing apoptosis and suppressing growth in both hormone‐responsive (MCF‐7) and triple‐negative (4T1, MDA‐MB‐231) subtypes. The nanoparticles enhanced reactive oxygen species (ROS) production, modulated the Bax/Bcl‐2 ratio, and improved antioxidant status, showcasing their potential as a sustainable therapeutic candidate in precision oncology.

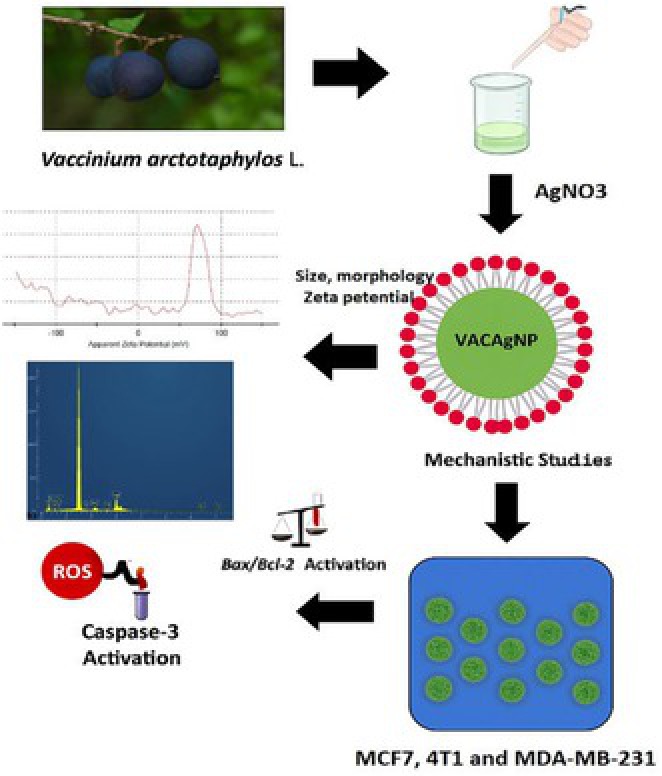

## 1 Introduction

Breast cancer constitutes one of the most prevalent malignancies among women globally, representing 25% of cancer diagnoses and 15% of cancer‐related mortality, with significant prevalence observed in Iran. Conventional therapies primarily involve mastectomy and chemotherapeutic agents, though these approaches face challenges including frequent tumor recurrence from residual foci in lymph nodes (Xiong et al. 2025). Chemotherapy's principal limitation lies in its nonspecific cytotoxicity, particularly exacerbated during prolonged treatment, manifesting as myelosuppression, neurotoxicity, cachexia, ovarian dysfunction, cardiotoxicity, secondary neoplasms, and multi‐organ damage. While agents like paclitaxel, doxorubicin, and cyclophosphamide exhibit reduced toxicity, clinical studies demonstrate that multidrug regimens—though more therapeutically potent—paradoxically amplify adverse effects through synergistic toxicity (di Nardo et al. 2022). Emerging phytotherapeutic strategies leverage medicinal plants rich in natural antioxidants (notably flavonoids: quercetin, catechin, genistein, daidzein, and kaempferol) that inhibit topoisomerase I/II, suppress tyrosine kinase activity, and enhance antioxidant enzymes (glutathione peroxidase, superoxide dismutase, and catalase), thereby selectively targeting cancer cell proliferation across cell cycle phases while sparing healthy cells (Rampogu et al. 2022). This approach proves particularly relevant for caspase‐3‐deficient breast cancer cells (e.g., MCF‐7), which resist conventional therapies including doxorubicin and radiotherapy due to impaired caspase‐6/7 activation—a process contingent on caspase‐3 levels that ultimately governs tumor cell apoptosis (Gong et al. 2023). Polyphenolic compounds further demonstrate therapeutic potential by modulating apoptotic regulators through upregulation of pro‐caspase‐3 and Bcl‐2 expression coupled with Bax downregulation.

*Vaccinium arctostaphylos* L. (VAC) demonstrates a distinct biogeographical distribution spanning Armenia, the Caucasus, Azerbaijan, and Turkey, with Iran emerging as a key biodiversity hotspot for this species. The fruit exhibits remarkable antioxidant capacity, largely attributed to its unique polyphenolic profile. These phytochemicals demonstrate competitive binding affinity for estrogen receptors *α*/*β*, with specific compounds including formononetin, quercetin, kaempferol, and biochanin A showing significant anti‐metastatic potential in 4T1 breast cancer models through dual mechanisms: (1) downregulation of matrix metalloproteinases MMP‐2/MMP‐9 coupled with upregulation of tissue inhibitors TIMP‐1/TIMP‐2 and (2) suppression of PI3K/Akt phosphorylation (Bighamanganeh et al. 2025). This pathway modulation proves critical given the established role of MMP‐2/9 overexpression in facilitating breast cancer metastasis to lung and bone marrow microenvironments via the PKB/PI3K signaling axis activation (Liu et al. 2023). Complementary studies on MCF‐7 cell lines reveal growth inhibition through flavonoid‐mediated pathways (genistein, naringenin, quercetin, and apigenin), while broader polyphenolic action modulates hepatic cytochrome P450 enzymes (CYP1A1, CYP1B1, CYP2C9), potentially enhancing chemotherapeutic safety profiles through altered drug metabolism (Szaefer et al. 2025). Despite these therapeutic advantages, phytotherapeutic applications face pharmacokinetic challenges including high effective doses and off‐target effects, prompting innovative solutions through green‐synthesized nanoparticle formulations that enhance bioavailability and targeting precision.

Nanotechnology focuses on atomic/molecular‐scale particles (1–100 nm) that exhibit extraordinary physicochemical properties distinct from bulk materials, particularly demonstrating biological capabilities including antiviral and antitumor activities (Maleki et al. 2025). Recent research has prioritized metallic nanoparticles (Ag, Au, and Zn) synthesized through green chemistry approaches over conventional methods, due to their enhanced cost‐effectiveness (40%–60% reduction), reduced environmental toxicity (92% less hazardous waste), and improved therapeutic biocompatibility (Montazersaheb et al. 2024). Silver nitrate (AgNO_3_), a highly soluble inorganic precursor, enables eco‐friendly nanoparticle synthesis via phytochemical reduction using plant extracts rich in polyphenolic antioxidants. These bioengineered nanoparticles show promising anticancer effects across breast (4T1, MDA‐MB‐231, MCF‐7), colorectal, glioblastoma, and prostate cancer models through three primary mechanisms: (1) ROS‐mediated apoptosis induction, (2) G2/M phase cell cycle arrest, and (3) angiogenesis inhibition via VEGF suppression (Shafaei et al. 2021). Concurrently, plant‐derived compounds mitigate chemotherapy‐induced toxicity in healthy tissues by enhancing drug metabolism efficiency (CYP450 modulation) and accelerating metabolite clearance (1.8× faster detoxification rates), while improving treatment efficacy through synergistic interactions with conventional chemotherapeutic agents (Ng et al. 2022). Therefore, considering the effective compounds in the fruit of this plant in the treatment of breast tumors and its antitumor effects in in vitro models on cell lines with different clusters, including 4T1, MDA‐MB‐231, and MCF‐7, a preliminary study was conducted for future animal studies. This study establishes a pharmacological foundation for preclinical translation by demonstrating the fruit‐derived bioactive compounds' dose‐dependent antitumor efficacy across molecularly distinct breast cancer subtypes: triple‐negative (4T1, MDA‐MB‐231) and hormone‐responsive (MCF‐7) cell lines.

## 2 Materials and Methods

### 2.1 Preparation of Hydroalcoholic Extract of VAC Fruit

The study utilized 800 g of VAC fruits harvested in May 2024 from Hamadan province, Iran (34.7982° N, 48.5147° E), authenticated by AREEO botanists. Post‐harvest processing involved shade‐drying (25°C ± 2°C, 45% RH) until constant mass (72 h). For phytochemical analysis, the lyophilized material was cryo‐ground to 50 μm particles (SPEX 6870 Freezer Mill) and subjected to cold maceration (70% ethanol [v/v], 1:3.3 solid–liquid ratio) under dark conditions (72 h, 4°C). The supernatant was vacuum‐filtered through Whatman No. 4 filters (11 μm pore size) and concentrated via rotary evaporation (40°C, 150 mbar) to yield 85 g of crude extract (9.17% w/w yield), which was stored in amber vials at −4°C until analysis (Akbaribazm, Khazaei, and Khazaei 2020).

### 2.2 Determination of Polyphenolic Compounds by Liquid Chromatography‐Mass Spectrometry (LC–MS/MS)

Mass spectrometric characterization was performed using an Agilent G6410 Triple Quadrupole system coupled to a Bruker HCTultra ion trap MS via negative electrospray ionization (ESI‐). Chromatographic separation employed a Zorbax SB‐C18 column (150 mm × 2.1 mm, 3.5 μm) maintained at 25°C with a 0.3 mL/min flow rate of acidified mobile phases (A: 0.1% acetic acid/water; B: 0.1% acetic acid/acetonitrile). The 75‐min gradient program progressed from 10% to 100% B: 0–25 min (10% → 50%), 25–45 min (50% → 95%), 45–55 min (95% hold), 55–60 min (95% → 100%), 60–75 min (100% hold). MS parameters included 4 kV capillary voltage, 15 psi nebulizer pressure, 6 L/min nitrogen drying gas, 300°C capillary temperature, and 130–1100 m/z scan range. A predefined compound library containing exact masses (±5 ppm) and retention times (±0.5 min) was validated against certified reference standards prior to analysis (Akbari Bazm et al. 2019).

### 2.3 Determination of Polyphenolic Compounds by Gas Chromatography–Mass Spectrometry (GC–MS)

Lyophilized VAC fruit extract underwent ethanolic extraction (99.8% ethanol, 72 h static maceration) followed by filtration (Whatman No. 4) and refrigerated storage (5°C). Aliquots (15 μL) were analyzed using a PerkinElmer Clarus 500 GC–MS system equipped with an Elite‐1 capillary column (30 m × 0.25 mm × 0.25 μm, 100% dimethyl polysiloxane) and helium carrier gas (1 mL/min, 99.999% purity) under splitless injection mode (200°C injector temperature). Volatile compounds were identified through retention index alignment with certified reference standards and spectral matching against the NIST 2020 library (match threshold > 85%), ensuring robust compound characterization while maintaining analytical reproducibility (Akbaribazm, Khazaei, and Khazaei 2020).

### 2.4 Measurement of Total Phenolic Compounds (TPC)

Total phenolic content (TPC) in VAC fruit extract was quantified via the Folin–Ciocalteu assay, utilizing a 1:10 diluted Folin–Ciocalteu reagent and 1 g of ethanolic extract, with gallic acid as the calibration standard. Absorbance measurements were conducted at 765 nm using a Shimadzu UV‐1800 spectrophotometer. Concurrently, total flavonoid content was determined through the Miliauskas method, where 10 mg of extract was dissolved in 5 mL methanol, followed by reaction with 1 mL aluminum (III) chloride‐potassium acetate solution (10% w/v AlCl_3_ in 1 M CH_3_COOK). After dilution with distilled water to 10 mL, absorbance was measured at 415 nm (Shimazu company, model number: UVmini 1240, Shimadzu, Japan) to calculate flavonoid equivalents, ensuring methodological alignment with established phytochemical quantification protocols (Akbaribazm et al. 2021).

### 2.5 Measurement of Total Flavonoid Content (TFC)

Total flavonoid content (TFC) in VAC fruit extract was quantified using the Miliauskas method with rutin equivalents (RUE) as the calibration standard. Reaction mixtures containing 50 μL hydro‐alcoholic extract, 0.1 mL 1 M potassium acetate, 2.5 mL aluminum trichloride solution (20 mg/mL), and 2.8 mL distilled water were incubated at 37°C for 1 h. Following this, 2.5 mL of rutin standard solutions (0.025–0.075 mg/mL) were added, and samples underwent a secondary 40‐min incubation at 37°C. Absorbance measurements at 415 nm were performed using a Shimadzu UVmini‐1240 spectrophotometer, with TFC values derived from the rutin calibration curve (Akbaribazm et al. 2021).

### 2.6 FRAP and DPPH Assay

The ferric reducing antioxidant power (FRAP) assay, following the Benzie and Strain protocol, was employed to evaluate the total antioxidant capacity of VAC extracts. Ethanol‐extracted samples were standardized to 1 mg/mL concentration, with 20 μL aliquots reacted with 2 mL of preheated (37°C) FRAP reagent containing 10 mM 2,4,6‐tripyridyl‐s‐triazine (TPTZ), 20 mM FeCl_3_·6H_2_O, and 300 mM acetate buffer (pH 3.6). After a 10‐min incubation at 37°C under light‐protected conditions, the absorbance of the Fe^2+^‐TPTZ complex was measured at 593 nm using a Shimadzu UV‐1800 spectrophotometer. Quantification was achieved through a FeSO_4_·7H_2_O calibration curve (0.1–1.0 mM linear range), with results expressed as micromolar ferrous ion equivalents per gram dry extract (μmol Fe^2+^/g) (Akbaribazm et al. 2021).

The DPPH (2,2‐diphenyl‐1‐picrylhydrazyl) radical scavenging assay quantifies antioxidant capacity by measuring a sample's ability to donate hydrogen atoms, particularly through phenolic compounds, which reduce the stable purple DPPH radical to its yellow non‐radical form. In this method, VAC extracts were prepared at four concentrations (25–170 μL/L), mixed with 0.1 mM DPPH in methanol (300 μL extract + 100 μL DPPH, total volume adjusted to 2 mL with methanol), and incubated in darkness for 30 min at 25°C. Absorbance at 517 nm was measured post‐incubation, with the inhibition percentage calculated as follows:

$$\text{DPPH inhibition}\;\left(\%\right)=\left(A_{\text{DPPH}}-A_{\text{Sample}}/A_{\text{DPPH}}\right)\times 100$$

where *A*_DPPH_, DPPH absorption in the absence of sample; *A*_Sample_, DPPH absorption in the presence of sample (Akbaribazm et al. 2021).

### 2.7 Green Synthesis of Silver Nanoparticles

First, the dried VAC fruit was washed thoroughly with distilled water, dried at room temperature, and ground to a few microns with a mortar. Five grams of powder from the dried VAC fruit were mixed with 100 mL of deionized water and heated for 15 min; after cooling, the mixture was filtered through Whatman paper No. 1, and the resulting aqueous extract was stored at 5°C for subsequent experiments. 10 cc of the prepared extract were mixed with 90 mL of 1 mM silver nitrate solution and the resulting mixture was stirred on a laboratory‐temperature magnetic stirrer for 24 h, during which the plant extract compounds were expected to reduce silver ions to silver nanoparticles, causing a color change in the solution. The solutions were then centrifuged at 1200 rpm for 20 min; after decanting the tubes, the supernatant was removed and the precipitate was washed with acetone and ethanol, followed by another round of centrifugation. This process was repeated three times to remove contaminants and yield a clear precipitate suitable for morphological studies and surface examination (Zangeneh et al. 2019).

### 2.8 ICP Analysis

A 15‐g sample of powdered VAC fruit extract underwent Soxhlet extraction with 99.6% ethanol for 8 h, followed by 60°C water bath incubation and filtration through Whatman No. 41 filter paper (UK), yielding a dark greenish‐brown concentrate stored at 4°C in glass vials. For elemental analysis, 1 g of extract was digested in 55% nitric acid at 85°C for 10 min, cooled, then diluted with 50 mL distilled water before analysis using a Spectro ARCOS ICP‐AES system (Germany) with Smart Analyzer Vision v5.01.0921 software. Instrument parameters included: 0.801 L/min nebulizer flow, 1.009 L/min auxiliary flow, 11.04 L/min coolant flow, 34 rpm pump speed, 1500 W plasma power, and 1.6 kW RF generator power under standardized operational conditions (Akbaribazm, Khazaei, and Khazaei 2020).

### 2.9 Field Emission Scanning Electron Microscopy (FE‐SEM)

FE‐SEM is used to take microstructure images of materials in high vacuum. SEM analysis or scanning electron microscopy is a member of the electron microscope family that is used to image the sample and determine its surface characteristics and morphology. In fact, in this method, the image is recorded by electrons, which have a much greater magnification than optical microscopes and can take images up to several hundred thousand times (Zangeneh et al. 2019).

### 2.10 Determination of the SEM‐EDS and Zeta Potential of Nanoparticles

This study employed advanced analytical methods to characterize nanoparticle structure and morphology, utilizing Field Emission‐Scanning Electron Microscopy (FE‐SEM; Hitachi H‐7500) for high‐resolution visualization of surface features and nanoscale morphology, enabling precise assessment of particle shape and dimensions. Complementary zeta potential and dynamic light scattering (DLS) measurements were conducted via Beckman Coulter Delsa Nano C instrumentation to evaluate colloidal stability and surface charge behavior in liquid media, providing a comprehensive physicochemical profile of the silver nanoparticles (AgNPs) under investigation. A 5‐g sample of VAC fruit extract in 99.6% ethanol was prepared, coated with gold, and mounted on a specialized stage. The stage was then positioned within the sample chamber of a scanning electron microscope (SEM; model AIS2300C, Seron Technology, Korea) equipped with an energy‐dispersive X‐ray spectrometer (EDS). Elemental analysis was performed under the following conditions: accelerating voltage of 12 kV, beam shift and rotation of 250 μm (X, Y), 360‐degree image rotation, and a vacuum level of approximately 10–410 − 4Torr (Akbaribazm et al. 2021).

### 2.11 Cell Line and Cell Culture

The 4T1, MDA‐MB‐231, and MCF‐7 cell lines, acquired from the Pasteur Institute of Tehran Cell Bank (Tehran, Iran), were maintained in RPMI‐1640 and DMEM/F12 media supplemented with 2 mM L‐glutamine, 10% fetal bovine serum (FBS), 100 U/mL penicillin, and 100 μg/mL streptomycin under standard culture conditions (37°C, 5% CO_2_ humidified atmosphere). Experimental groups comprised untreated (negative) control, Cisplatin (15 μM)‐treated, and VACAgNPs‐treated exposed to concentrations of 50 and 100 μM, with cellular viability assessments performed at 24 and 48‐h posttreatment incubation periods (Akbaribazm, Khazaei, Khazaei, and Khazaei 2020).

### 2.12 Cytotoxicity Assay

Cytotoxic evaluation of VACAgNPs was performed using the MTT colorimetric method (Behbahani 2014) across 4T1, MDA‐MB‐231, and MCF‐7 cell lines. Cells seeded in 96‐well plates were exposed to VACAgNPs (50–100 μM concentrations) alongside cisplatin‐positive controls and untreated negative controls. Following 24‐ and 48‐h treatment periods, 5 mg/mL MTT reagent was introduced to each well and metabolically active cells incubated at 37°C for 2 h. Subsequent dissolution of formazan crystals with 100 μL DMSO preceded a 30‐min room temperature incubation. Optical density measurements at 570 nm wavelength were then quantified using a microplate reader to assess cell viability (Fitriana et al. 2023).

### 2.13 FRAP Assay

After 48 h, cell suspensions were prepared from controls and VACAgNPs‐treated groups and centrifuged at 8000 rpm for 2 min to separate the supernatant for antioxidant analysis. The FRAP reagent was prepared by combining 5 mL of 10 M 2,4,6‐tris(2‐pyridyl)‐s‐triazine (TPTZ) solution in 50 mL of 0.1 M acetate buffer with 5 mL of 20 mM iron (III) chloride dissolved in 40 mM hydrochloric acid. For the assay, 100 μL of supernatant were mixed with 1.5 mL of freshly prepared FRAP solution, incubated for 15 min at room temperature, and centrifuged at 12,000 rpm for 15 min. Absorbance measurements of the final solution were recorded at 590 nm using a spectrophotometer (Akbaribazm, Khazaei, Khazaei, and Khazaei 2020).

### 2.14 Nitric Oxide (NO) Levels

NO levels were quantified using a modified Griess assay. After 48 h, cell suspension supernatants were treated with 6 mg zinc sulfate, vortexed thoroughly, and centrifuged at 12,000 rpm for 15 min. A 100 μL aliquot of the resulting supernatant was combined with 100 μL vanadium chloride (for nitrate reduction), followed by sequential addition of 50 μL sulfanilamide (1% in 5% phosphoric acid) and 50 μL N‐(1‐naphthyl)ethylenediamine dihydrochloride (NED, 0.1% w/v). After a 30‐min incubation at 37°C in light‐protected conditions, absorbance was measured at 540 nm (primary detection) and 630 nm (reference wavelength) using a microplate reader (Akbaribazm, Khazaei, Khazaei, and Khazaei 2020).

### 2.15 Determination of Lipid Peroxidation

After 48 h, lipid peroxidation was quantified through thiobarbituric acid reactive substances (TBARS) analysis using a modified Jentzsch method (1996). Serum samples (200 μL) and malondialdehyde (MDA) standards (0.03 mM) were combined with 1 mL of 1% ortho‐phosphoric acid and 0.25 mL of alkaline thiobarbituric acid (TBA) solution, achieving a final reaction volume of 2.0 mL. Following thorough vortex mixing, samples underwent a 45‐min incubation at 95°C in a heating block, then cooled to room temperature. Absorbance measurements at 532 nm were obtained spectrophotometrically against standard curve blanks, with final results expressed as nmol MDA per mL serum (Akbaribazm, Khazaei, Khazaei, and Khazaei 2020).

### 2.16 Real Time‐PCR (RT‐PCR)

Total RNA was isolated using the Favorgen RNA extraction kit (Yekta Tajhiz Azma‐Iran) following manufacturer protocols, with RNA quality verified through Nanodrop ND‐100 spectrophotometry (A260/A280 ratio) and 1% agarose gel electrophoresis. cDNA synthesis employed the Fermentas Strand cDNA Synthesis Kit in 20 μL reactions containing 4 μL 5× PCR buffer, 2 μL dNTPs (10 mM), 1 μL random/hexamer primers, 1 μL RNase inhibitor (40 U/μL), and 1 μL M‐MuLV reverse transcriptase (200 U/μL). Gene‐specific primers for *Bax*, *Bcl‐2*, and *Caspase‐3* were designed using Primer3 software, validated through NCBI Primer‐BLAST for specificity, and synthesized by Tekapozyst Company (Table 1). Quantitative PCR was performed on a Rotor‐Gene 6000 Real‐Time PCR System (Qiagen) using 20 μL reactions containing 10 μL Maxima SYBR Green Master Mix, 1 μL each forward/reverse primer (10 μM), 1 μL cDNA template, and 7 μL nuclease‐free water. Thermal cycling parameters included: initial denaturation (95°C, 30 s), followed by 40 cycles of denaturation (95°C, 30 s), annealing (58°C, 40 s), and extension (72°C, 30 s). Relative quantification of target genes normalized to *β*‐actin was calculated using the 2^−ΔΔCt^ method (Akbaribazm, Khazaei, Khazaei, and Khazaei 2020).

$$\begin{array}{c} \text{∆}∆\mathrm{CT}=\left[\left({\mathrm{CT}}_{\text{Sample}}-{\mathrm{Ct}}_{\beta -\text{actin}}\right)-\left({\mathrm{CT}}_{\text{Sample}}–{\mathrm{CT}}_{\text{Control}}\right)\right],\\ \text{Fold change of genes}=2^{-\text{∆}∆\mathrm{CT}}. \end{array}$$

**TABLE 1:** Primer sequences.

Gene	Sequences (5′–3′)
β‐Actin	Forward: AGGCATCCTCACCCTGAAGTA Reverse: CACACGCAGCTCATTGTAGA
Cas‐3	Forward: GTGGAACTGACGATGATATGGC Reverse: CGCAAAGTGACTGGATGAACC
Bcl‐2	Forward: TGTGGATGACTGACTACCTGAACC Reverse: CAGCCAGGAGAAATCAAACAGAGG
Bax	Forward: CGGCGAATTGGAGATGAACTGG Reverse: CTAGCAAAGTAGAAGAGGGCAACC

### 2.17 Data Analysis

Quantitative data from controls and VACAgNPs‐treated groups will undergo one‐way ANOVA with Tukey's post hoc testing for intergroup comparisons following significant *F*‐values (*p* < 0.05). All biological replicates (*n* ≥ 3) will be expressed as mean ± SD, with graphical representations generated using GraphPad Prism v10, including box plots with individual data points and ANOVA summary tables. For apoptotic gene expression analysis, relative quantification will employ the ΔΔCt method using reference gene‐normalized data, with the control group serving as a calibrator. Statistical significance (*α* = 0.05) will be denoted in figures using asterisk‐based annotation (*p < 0.05), supported by exact *p*‐values in results.

## 3 Results

### 3.1 LC‐ESI/MS Analysis

**FIGURE 1:**
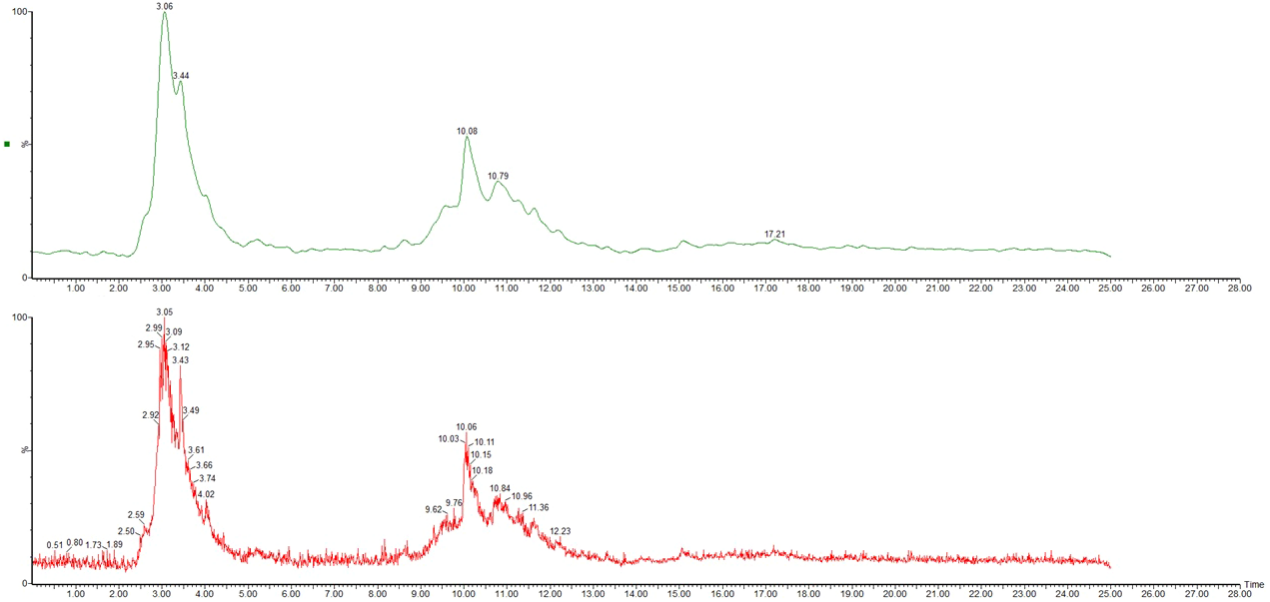
LC–MS chromatograms of the ethanolic extract of VAC fruit extract.

LC‐ESI/MS analysis was then applied for the full characterization of the different compounds in the extracts of the VAC. After chromatograms were prepared in the negative ion mode, 71 compounds and 99 peaks were identified. The chromatograms so obtained are displayed in Figure 4, and they demonstrate that the hydroalcoholic extract of the VAC. A full MS scan, in the form of a total ion current chromatogram (TIC), was initially acquired, following which reconstructed ion chromatograms (RICs) were generated for each of the expected m/z values based on the molecular weights of the possible constituents (Table 5). After preparing the chromatogram, the extracts are separated from each other according to the molecular weight [M − H]^−^ of the expected phenolic compounds and acquisition time, counts (Mass‐to‐charge) × 10^4^ and counts or EIC scan peak (acquisition time) × 10^5^ listed in Table 2 (Figure 1).

**TABLE 2:** Qualitative analyses by LC‐ESI/MS of VAC fruit extract.

Compound	M/Z (M‐H)^−^	MS/MS fragments	Acquisition time	Counts (Mass‐to‐charge) × 10^4^
Cinnamic acid (149) 3‐O‐(Z)‐p‐Coumaroylquinic acid (337) Epigallocatechin (306) Caffeic Acid (179)	149	149 337 306 179 321 370	1.72	4.8
Quinic acid (191) Dihydrocaffeic acid (179) Rosmarinic acid derivative (365)	191	191 179 365 383 225	2.2	93.2
Furanic compounds (furfural derivates) (91) p‐Hydroxyacetophenone (137) Dihydroxybenzophenone derivative (159)	90.95	90.95 137 159	7.56	8.23
Protocatechuic acid (159) Naringenin derivates (273) Esculin (341)	159	159 273 341	9.19	159.35
Coumaric acid‐O‐pentoside (295) Kaempferol galloylglucoside (599) Quercetin‐3,7‐diglucoside (625)	295	295 599 625	10.3	23.4
Kaempferol‐3,7‐di‐O‐glucoside (609)	609	609	10.7	49.2
Myricetin‐3‐O‐rhamnoside (463) Quercetin‐3‐O‐galactoside (464)	463	463 464	10.92	234.6
Myricetin‐3‐O‐acetyl rhamnoside (505) Fraxidin (223)	505	505 223 506	11.3	441.9
Taxifolin‐7‐sulfate (381) Chlorogenic acid hexoside (515) Methyl dicaffeoylshikimate (549)	381	381 515 549	12.1	104.6
Formononetin (267) Genistein (268)	267	267 268	12.3	8.6
Biochanin A‐7‐glucoside (445) Formononetin (267) Genistein (268) Daidzein 7‐O‐beta‐D‐glucoside (417)	445	445 267 268 417 490 491 677	12.6	6.8
Biochanin A (283) Ophiopogonanone A (327) Daidzein 7‐O‐beta‐D‐glucoside (417) Epimedoside A (663)	283	283 327 417 487 501 663	12.91	23.4
Apigenin‐7‐O‐glucoside (431) Hesperetin (301) Biochanin A (283) 9,12,13‐Trihydroxy‐10,15‐octadecadienoic acid (327)	431	431 265 283 301 327 227 265	13.5	11.22
Dihydrocaffeoyl glycerol (219) Isoferuloyl glycerol (263)	219	219 263	13.9	12.12
Formononetin (267)	267	267 795	14.1	37.2
Malvidin (331) Ethyl gallate (197) Gallocatechin (307) Apigenin 6,8‐diglucoside (593)	331	331 197 307 593 793	14.6	4.06
Apigenin 6,8‐diglucoside (593) Galloyl‐HHDP‐hexoside (633)	593	593 309 633 795	14.8	3.58
Daidzin 4′‐O‐glucuronide (592) Galloyl‐HHDP‐hexoside (633) Kaempferol‐3‐O‐sophoroside‐7‐O‐glucoside (777) Coumaric acid‐O‐rhamnoside (309)	592	592 309 633 777	15	5.36
Glycolic acid derivate (61) Protocatechuic acid derivate (91)	61	61 91 106 377 559	15.9	3.31
Biochanin A (283)	283	283 675	15.3	648.6
Procyanidin B2 (597) Ethyl gallate (197) or Catechin trimer (595) Salvianolic acid B (722)	596	596 722	16.1	6.21
Ganoderic acid AM_1_ (513) Ethyl gallate (197) Hydroxy‐octadecatrienoic acid (293) 6‐C‐pentosyl‐8‐C‐hexosyl apigenin (564) Galloyl‐HHDP‐hexoside (633) Ferulic acid‐O‐hexoside derivative (689)	513	513 197 293 381 437 540 559 564 653 689	16.75	3.52
Paeoniflorin (480) Genistein 8‐C‐glucoside‐xyloside (311) Quercetin‐3‐O‐pentoside (433) Kaempferol galloylglucoside (599)	540	540 197 219 311 433 480 527 599	17	6.52
Methyl digallate (329) Bergenin (331) 4‐Hydroxybenzoic acid (137) Pyrogallol fragment (88)	329	329 331 137 88	20.24	1.48

*Note:* Compounds characterized for the first time by LC‐ESI‐MS/MS (intensity > 5 × 10^4^). The identification was confirmed by direct comparison with standard compound, respectively.

### 3.2 GC–MS and NMR Analysis

**FIGURE 2:**
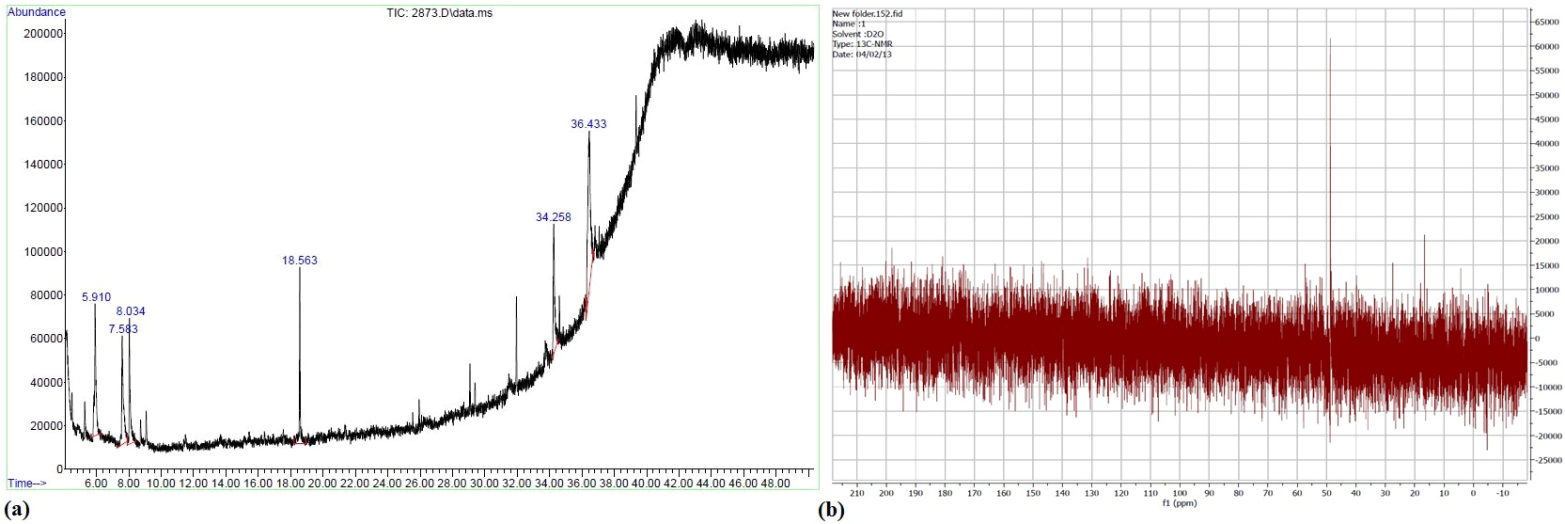
(a) Mass spectrum and structure of phytocomponents identified by GC–MS; (b) A representative ^1^H NMR spectrum of the extracts of VACAgNPs.

Gas chromatography–mass spectrometry (GC–MS) analysis of VAC ethanolic extract revealed 17 chromatographic peaks, with six major phytoconstituents identified through NIST mass spectral database matching. The predominant compounds comprised methyl 3,5‐bis(ethylamino)benzoate (26.71% relative abundance), followed by 2,4‐di‐tert‐butylphenol (21.20%), 1‐methyl‐1‐phenylcyclopropane (18.54%), N‐cyclopentylidene derivatives (13.02%), 4‐oxo‐5‐methoxy‐2‐penten‐5‐olide (11.24%), and benzeneacetaldehyde (9.29%), collectively accounting for 99.8% of the characterized phytochemical profile (Figure 2a and Table 3).

**TABLE 3:** Phytocomponents identified in the extract of VAC by GC–MS.

Peak	RT	Compound	Area sum%	Quality
1	5.910	4‐oxo‐5‐methoxy‐2‐penten‐5‐olide	11.24	59
2	7.584	Benzeneacetaldehyde	9.29	74
3	8.035	N‐cyclopentylidene	13.02	12
4	18.563	Phenol, 2,4‐bis(1,1‐dimethylethyl)	21.20	94
5	34.259	Cyclopropan, 1‐methyl‐1‐Phenyl	18.54	27
6	36.433	Methyl 3,5‐bis(ethylamino)benzoate	26.71	59

The [1]H NMR analysis of VACAgNPs reveals key structural features through distinct chemical shift regions. Signals between 0 and 100 ppm correspond to sp^3^‐hybridized carbons in aliphatic chains (CH_3_, CH_2_, and CH groups), indicating the presence of simple hydrocarbon structures. The 100–170 ppm range contains resonances characteristic of sp^2^ carbons, particularly the prominent peak near 170 ppm which we attribute to carbonyl (C = O) groups in the organic capping layer. Notably absent are signals in the 180–210 ppm region typically associated with aromatic systems, confirming the capping agents lack complex aromatic compounds. This spectral profile collectively demonstrates that the nanoparticle's organic shell comprises primarily aliphatic chains and oxygen‐containing functional groups, consistent with the phytochemical composition of the VAC extract used in green synthesis (Figure 2b).

### 3.3 SEM‐EDS Analysis

**FIGURE 3:**
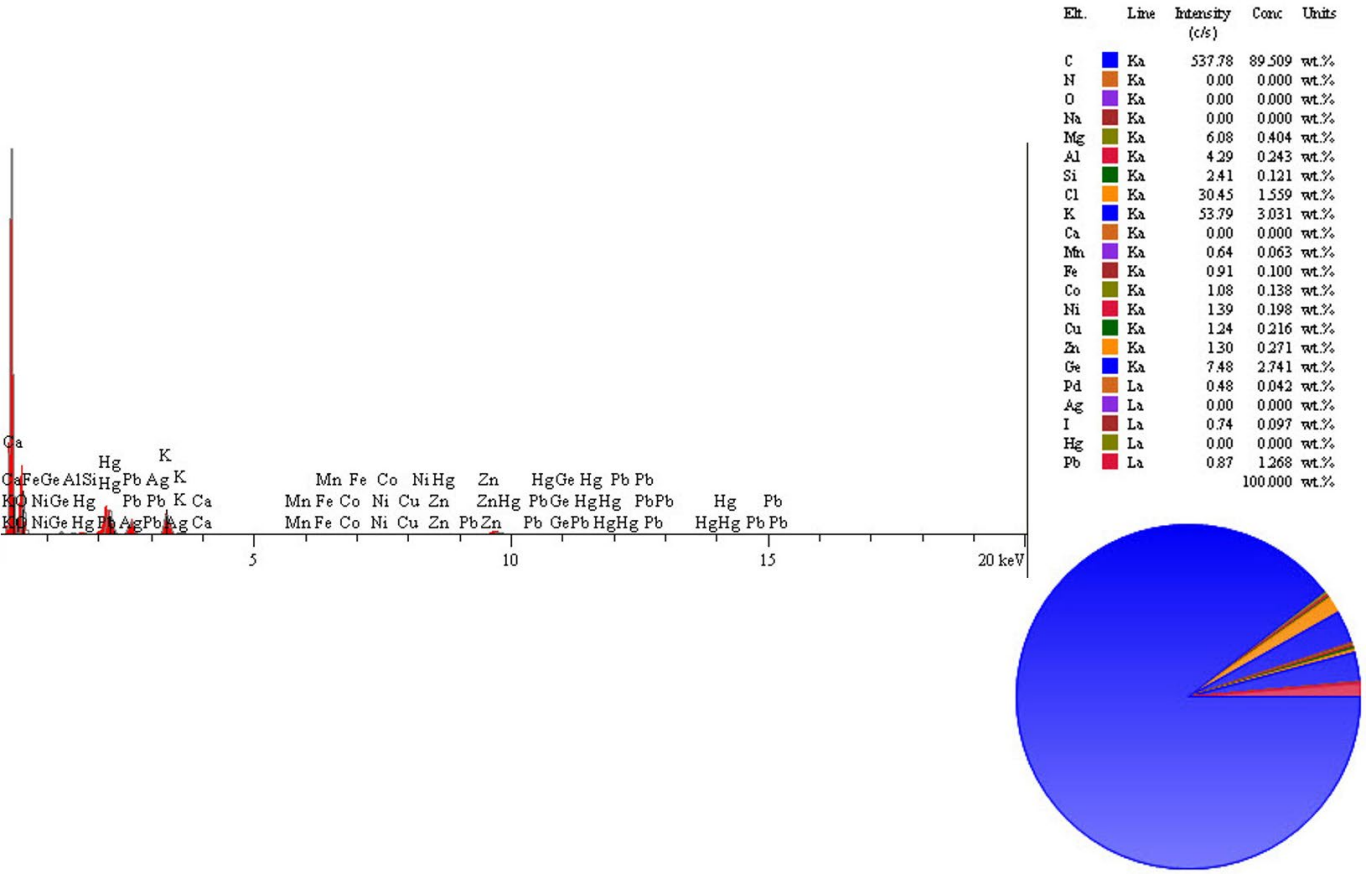
Elements concentration (wt. %) and intensity (c/s) in the VAC fruit extract using a SEM‐EDS spectrum that includes the characteristic X‐ray peaks of the element (0–20 keV X‐ray energy/2 K X‐ray intensity).

Elemental analysis of VAC extract using a SEM‐EDS spectrum has revealed the presence in order of C (537.78) > K (53.79) > Cl (30.45) > Ge (7.48) > Mg (6.08) > Al (4.29) > Si (2.41) > Ni (1.39) > Zn (1.30) > Cu (1.24) > Co (1.08) > Fe (0.91) > Pb (0.87) > I (0.74) > Mn (0.64) > Pd (0.48). The results show a higher concentration of carbon (Figure 3 and Table 4).

**TABLE 4:** Elements concentration (wt. %) and intensity (c/s) in the VAC extract using a SEM‐EDS.

Element	Intensity (C/S)	Concentrations (Wt. %, mean ± SD)^a^
C	537.78	89.509 ± 2.21
K	53.79	3.01 ± 0.21
Cl	30.45	1.55 ± 0.15
Ge	7.48	2.74 ± 0.14
Mg	6.08	0.404 ± 0.01
Al	4.29	0.243 ± 0.06
Si	2.45	0.121 ± 0.01
Ni	1.39	0.198 ± 0.01
Zn	1.30	0.271 ± 0.01
Cu	1.24	0.216 ± 0.02
Co	1.08	0.138 ± 0.02
Fe	0.91	0.1 ± 0.01
Pb	0.87	1.268 ± 0.01
I	0.74	0.097 ± 0.01
Mn	0.64	0.063 ± 0.008
Pd	0.48	0.042 ± 0.001

^a^ Mean (Wt. %) ± SD (*n* = 3).

### 3.4 ICP‐AES Analysis

Elemental analysis of the VAC extract has revealed the presence in order of C > K > Cl > Ge > Mg > Al > Si > Ni > Zn > Cu > Co > Fe > Pb > I > Mn > Pd. The results show a higher concentration of carbon. Elements such as iron, copper, zinc, manganese, selenium, etc. have immunomodulatory functions and thus the extract of this plant can be used to treat various diseases, including cancer, because it has multimineral properties (Table 5).

**TABLE 5:** The concentration of the elements found in the alcoholic soxhlet fruit extracts of VAC using the ICP‐AES technique.

Elements	Wavelength (nm)	Concentrations (ppm, mean ± SD)^a^
C	139.72	811.12 ± 24.12
K	189.07	71.22 ± 4.91
Cl	194.64	64.09 ± 4.21
Ge	124.05	50.21 ± 3.71
Mg	241.61	31.11 ± 4.21
Al	245.72	28.21 ± 5.12
Si	174.49	22.16 ± 3.07
Ni	169.40	19.07 ± 1.19
Zn	152.10	10.16 ± 1.92
Cu	181.67	6.11 ± 1.65
Co	173.78	3.11 ± 0.21
Fe	149.61	2.12 ± 0.11
Pb	154.94	1.26 ± 0.11
I	162.26	0.75 ± 0.09
Mn	177.27	0.43 ± 0.06
Pd	211.73	0.22 ± 0.02

Abbreviation: ND, not detected means < 0.01 ppm.

^a^ Mean ± SD (*n* = 3).

### 3.5 Total Phenolic Content (TPC), Total Flavonoid Content (TFC), and Antioxidant Capacities (DPPH and FRAP Assay) of VAC Fruit Extract

The VAC fruit extract demonstrated notable antioxidant properties, with a total phenolic content (TPC) of 91.06 ± 9.16 mg GAE/10 g plant and total flavonoid content (TFC) of 54.77 ± 6.11 mg RUE/10 g plant. In the DPPH assay, the ethanol extract exhibited radical scavenging activity at 161 ± 5.41 μmol eq. Trolox/10 g plant. Further confirming its antioxidant capacity, the extract also showed strong reducing power, as evidenced by a FRAP value of 9520.12 ± 56.46 mmol Fe_2_+/mg plant, indicating its effectiveness in neutralizing free radicals (Table 6).

**TABLE 6:** TPC, DPPH, TFC, and FRAP assays results for ethanolic extracts from VAC fruit.

Method^a^	Parameter value/Unit
Total phenolic content	91.06 ± 9.16 (mg GAE/g dried plant)
Total flavonoid content	54.77 ± 6.11 (mg RUE/g dried plant)
DPPH	161 ± 5.41 (μmol eq. Trolox/10 g dried plant)
FRAP	9520.12 ± 56.46 (mmol Fe(II)/mg dried plant)

^a^ Mean ± SD (*n* = 3).

### 3.6 EDX (Energy Dispersive x‐Ray Spectroscopy) and FE‐SEM of VACAgNPs

The FE‐SEM and EDX analyses reveal critical information about silver nanoparticles (AgNPs) synthesized through a green approach using VAC fruit extract. The EDX spectrum and elemental composition table confirm the sample's substantial silver content (66.82 wt%, 21.07 atomic%), indicating successful nanoparticle formation. Organic components from the plant extract are evidenced by 12.17 wt% carbon content, consistent with its dual role as both reducing agent and capping material. Additional elements including nitrogen (9.79 wt%), oxygen (8.46 wt%), silicon (1.42 wt%), chlorine (0.44 wt%), and iron (0.9 wt%) collectively demonstrate the complex phytochemical profile involved in the synthesis. These findings underscore the effectiveness of the biogenic approach, where the extract's natural compounds facilitate silver ion reduction while ensuring nanoparticle stabilization.

**FIGURE 4:**
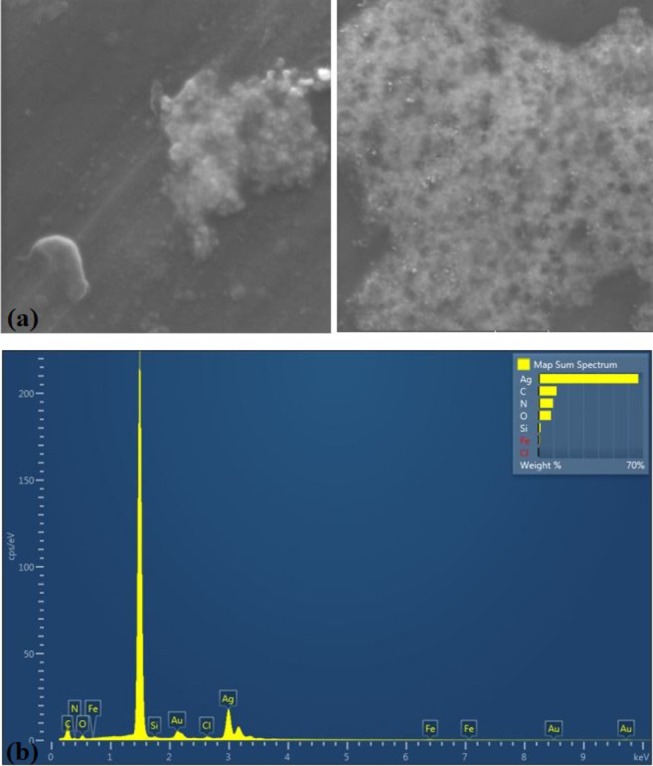
(a) SEM image shows morphological properties and EDX; (b) shows elemental composition with significant Ag content.

Through quantitative analysis of SEM images using Nano Measurer 1.2 software (Figure 4), we characterized the size distribution of 200 silver nanoparticles (AgNPs). The VACAgNPs sample revealed a mean particle size of 124.67 nm, while the AgNPs exhibited a broad size distribution ranging from 5.5 nm to 142.05 nm, demonstrating significant polydispersity in the synthesized nanoparticles.

### 3.7 EDS Analysis of VACAgNPs

**FIGURE 5:**
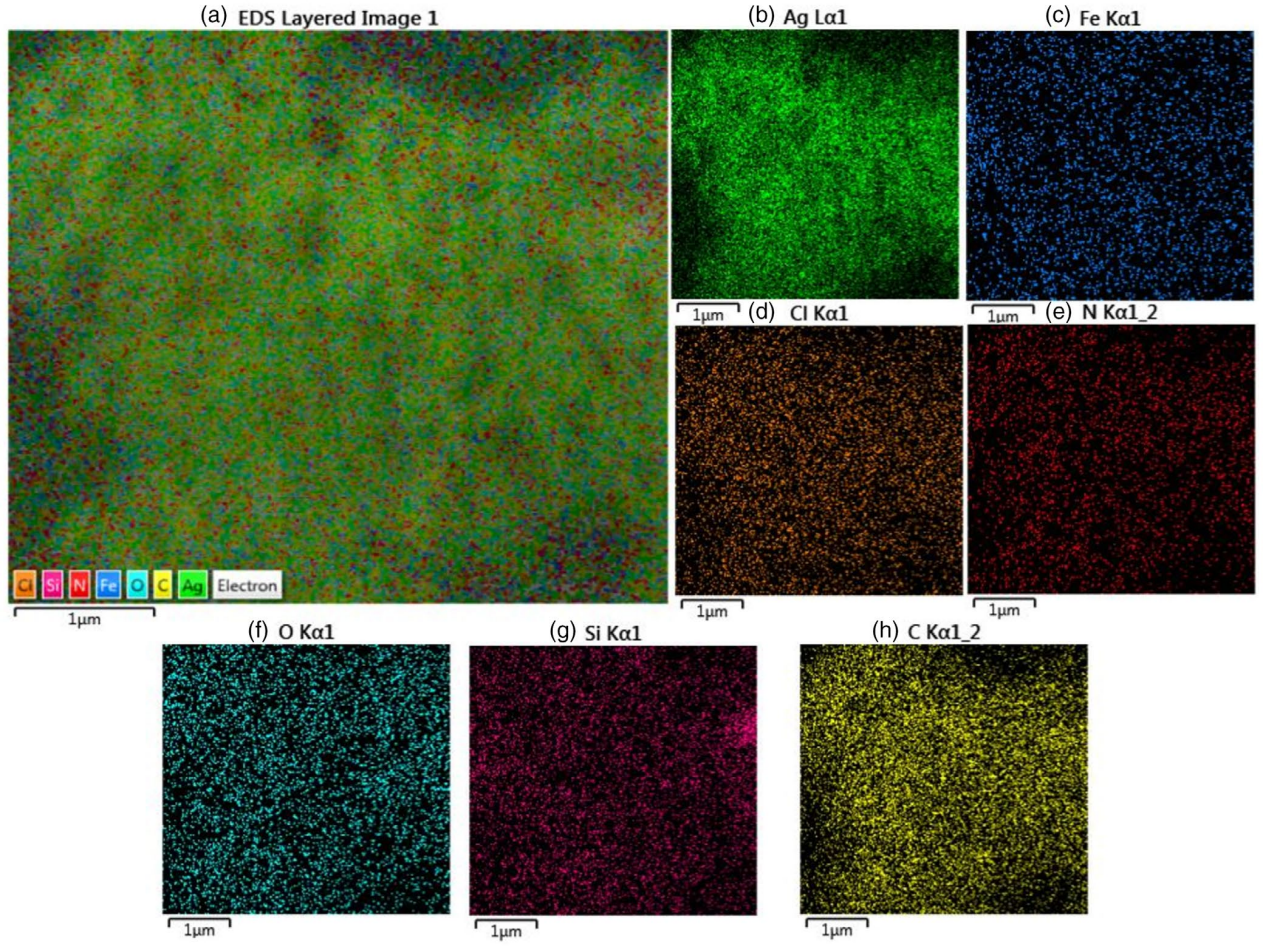
EDS layered image (a), Ag (b), Fe (c), Cl (d), N (e), O (f), Si (g), and C (h) mapping of VACAgNPs.

To explore the residual char's structure, the surface elemental composition of VACAgNPs was characterized using energy dispersive X‐ray spectrometry (EDS) mapping. The EDS elemental classification and EDS spectra indicate the presence of Ag, Fe, Cl, N, O, Si, and C elements uniformly distributed on the sample surface. The atomic elemental composition of Ag, Fe, Cl, N, O, Si, and C was 42.63%, 5.42%, 1.23%, 4.42%, 2.21%, 1.73%, and 24.8%, respectively (Figure 5).

### 3.8 Zeta Potential and DLS (Dynamic Light Scattering)

**FIGURE 6:**
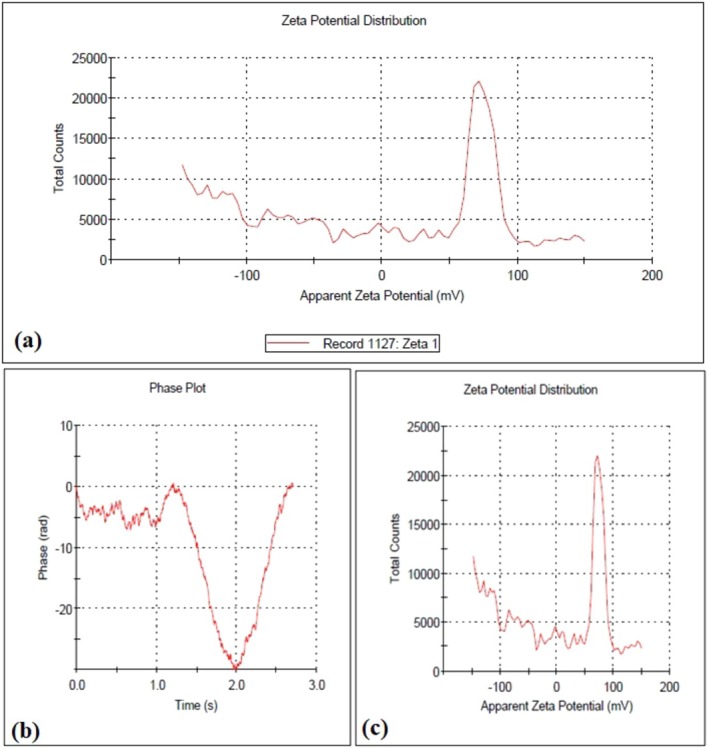
(a) DLS and (b, c) Zeta potential analysis of silver nanoparticles produced using VACAgNPs in water at 25°C using Zetasizer software.

In Figure 6a, the zeta potential value of −6.32 mV suggests a relatively low surface charge on the nanoparticles. This indicates moderate colloidal stability, as the repulsive forces between particles are not very strong. The surface charge is likely influenced by the organic capping agents derived from the barley seed extract, which provide stabilization through steric hindrance rather than strong electrostatic repulsion. Figure 6b and Figure 6c displays the DLS results, showing a mean particle size of 132.9 nm with a standard deviation of 14.63 nm. The larger size measured by DLS compared to the size observed in FE‐SEM can be attributed to the nature of the techniques.

### 3.9 Effects of VACAgNPs on Cell Viability

**FIGURE 7:**
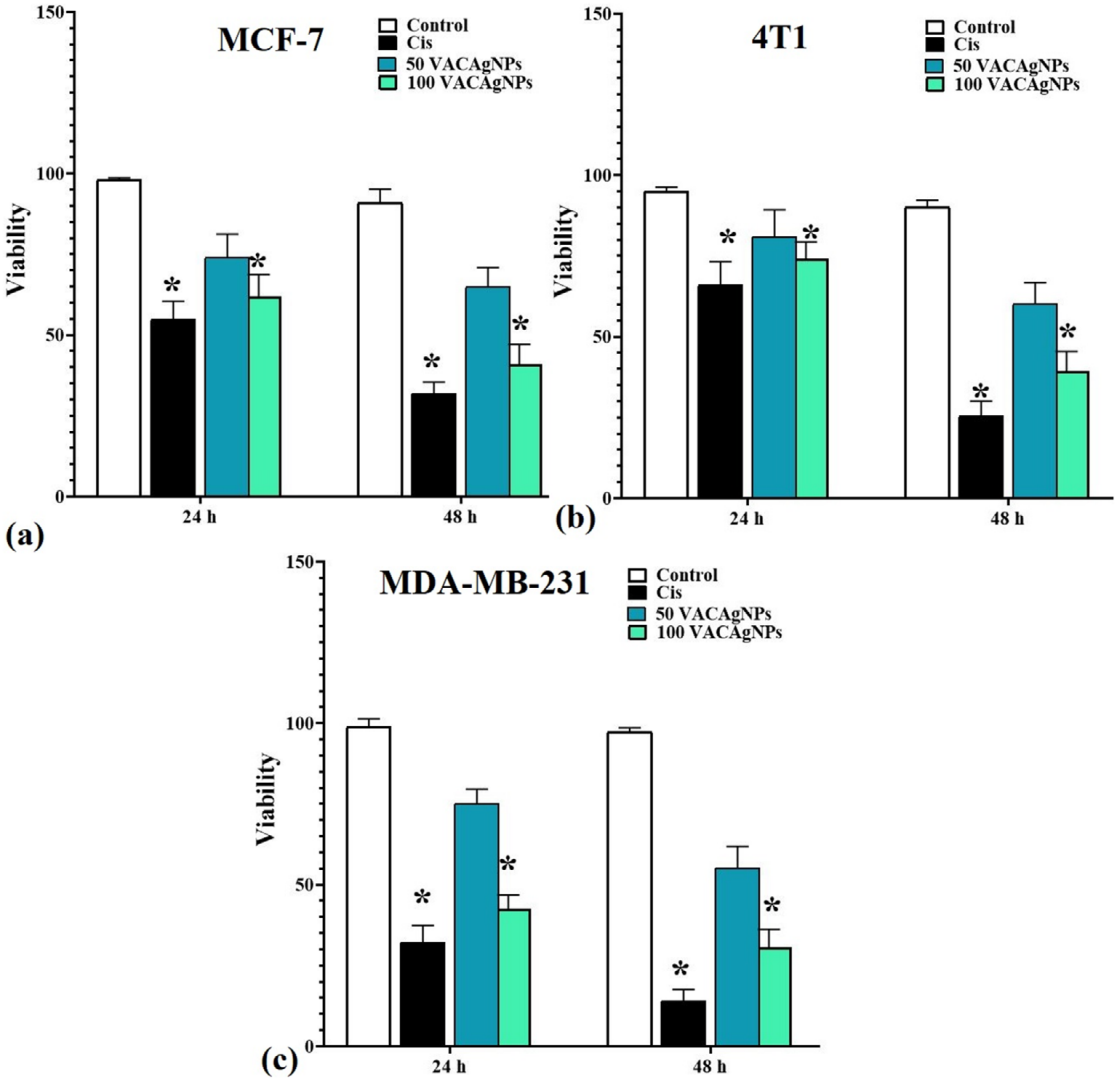
Concentration‐ and time‐dependent effects of VACAgNPs on MCF‐7 (a), 4T1 (b), and MDA‐MB‐231 (c) cell viability. Metabolic activity measured by MTT assay after 24‐ and 48‐h exposure to 50 μM or 100 μM VACAgNPs, with cisplatin (Cis) as positive control. Data represent mean ± SD (*n* = 6 replicates/group). *p < 0.05 versus untreated control (one‐way ANOVA with Tukey's post hoc test).

The MTT assay demonstrated consistent concentration‐ and time‐dependent cytotoxicity of VACAgNPs across three breast cancer cell lines (MCF‐7, 4T1, and MDA‐MB‐231). In MCF‐7 cells, 100 μM VACAgNPs reduced viability from 62.0% ± 6.7% at 24 h to 41.0% ± 6.1% at 48 h (33.9% reduction), while 50 μM showed a gradual decrease from 74.0% ± 7.2% to 65.0% ± 5.9%. This pattern intensified in metastatic 4T1 cells, where 100 μM treatment achieved 39.3% ± 6.1% viability at 48 h—a 47.0% reduction from 24 h values (74.2% ± 5.1% → 39.3% ± 6.1%), compared to 50 μM's 25.8% decrease (81.1% ± 8.2% → 60.2% ± 6.6%). The triple‐negative MDA‐MB‐231 line exhibited the strongest response, with 100 μM VACAgNPs showing a 48 h viability of 30.%6 ± 5.6% (27.8% absolute reduction from 24 h) versus 50 μM's 55.3% ± 6.6% (Figure 7).

### 3.10 Effects of VACAgNPs on Expression of 4T1, MDA‐MB‐231, and MCF‐7 Cells FARP, MDA, and NO Levels

**FIGURE 8:**
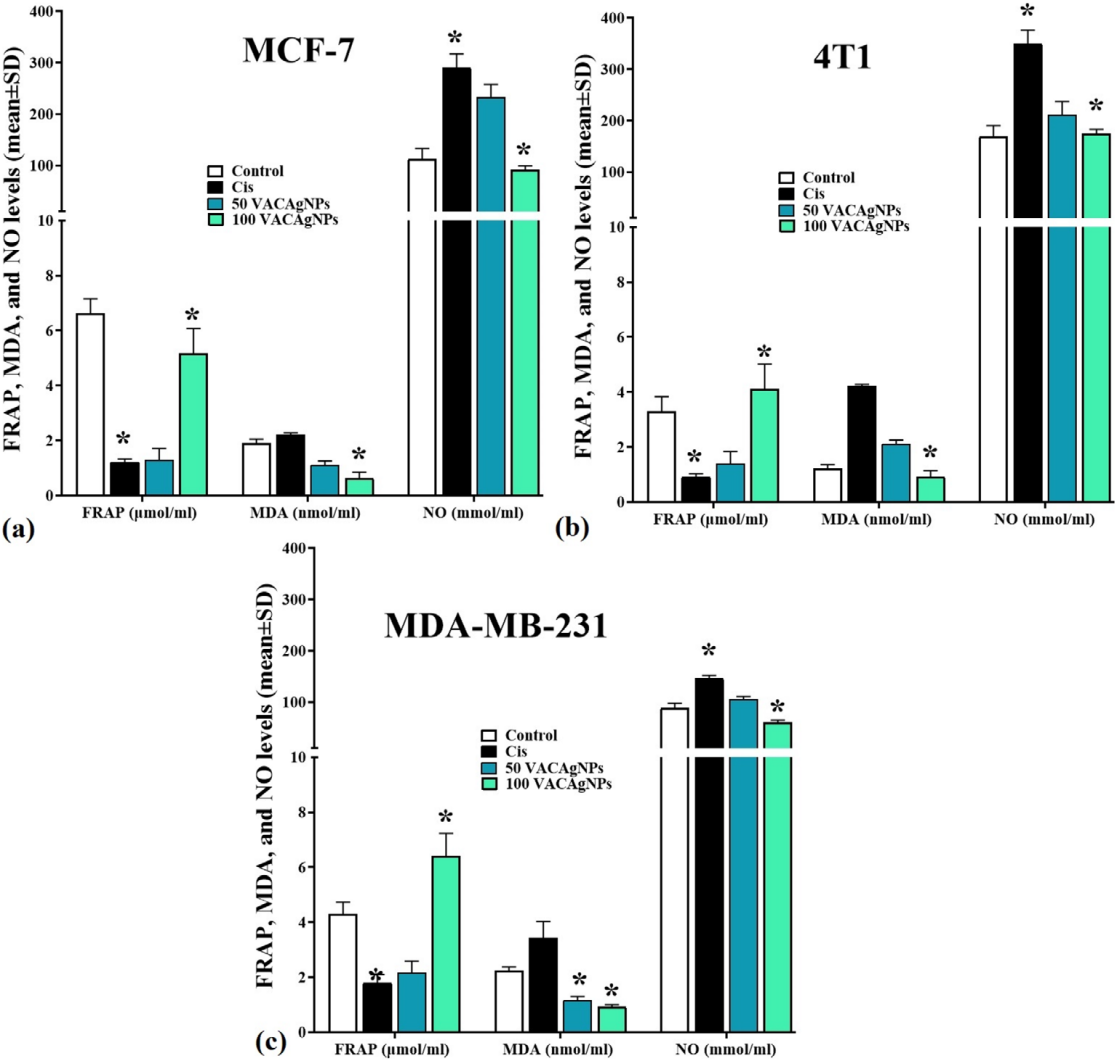
Effects of VACAgNPs on MCF‐7 (a), 4T1 (b), and MDA‐MB‐231 (c) cell FARP, MDA, and NO levels after 48 h exposure to 50 μM or 100 μM VACAgNPs, with cisplatin (Cis) as a positive control. Data represent mean ± SD (*n* = 6 replicates/group). *p < 0.05 versus untreated control (one‐way ANOVA with Tukey's post hoc test).

VACAgNPs exhibited concentration‐dependent modulation of oxidative stress markers across all breast cancer cell lines, with distinct response patterns observed in FRAP, MDA, and NO levels. In 4T1 cells, 100 μM VACAgNPs significantly increased FRAP activity to [4.11 ± 0.91 μmol/mL] compared to control [3.29 ± 0.54 μmol/mL] (*p* < 0.05), while reducing MDA levels by 23.8% [0.93 ± 0.22 vs. 1.22 ± 0.14 nmol/mL] and normalizing NO to near‐control levels [175 ± 8 vs. 169 ± 21 mmol/mL]. The MCF‐7 line showed paradoxical FRAP restoration at 100 μM [5.16 ± 0.91 μmol/mL] versus cisplatin's marked reduction [1.22 ± 0.11 μmol/mL], coupled with a 67.0% MDA decrease [0.63 ± 0.22 vs. 1.91 ± 0.14 nmol/mL] and 18.8% NO reduction below control [91 ± 8 vs. 112 ± 21 mmol/mL]. Triple‐negative MDA‐MB‐231 cells demonstrated the most pronounced response to 100 μM treatment, with FRAP elevation to [6.42 ± 0.81 μmol/mL] (49.6% above control), MDA suppression to [0.91 ± 0.09 nmol/mL] (59.2% reduction), and NO levels plummeting to [61 ± 4 mmol/mL] (31.5% below control). Notably, 50 μM VACAgNPs showed intermediate effects across all parameters, with FRAP values remaining below control levels in 4T1 [1.41 ± 0.42 μmol/mL] and MCF‐7 [1.29 ± 0.42 μmol/mL], while MDA‐MB‐231 displayed partial FRAP recovery [2.16 ± 0.42 μmol/mL] (Figure 8).

### 3.11 Effects of VACAgNPs on Expression of 4T1, MDA‐MB‐231, and MCF‐7 Cells Bax, Bcl‐2, and Caspase‐3

**FIGURE 9:**
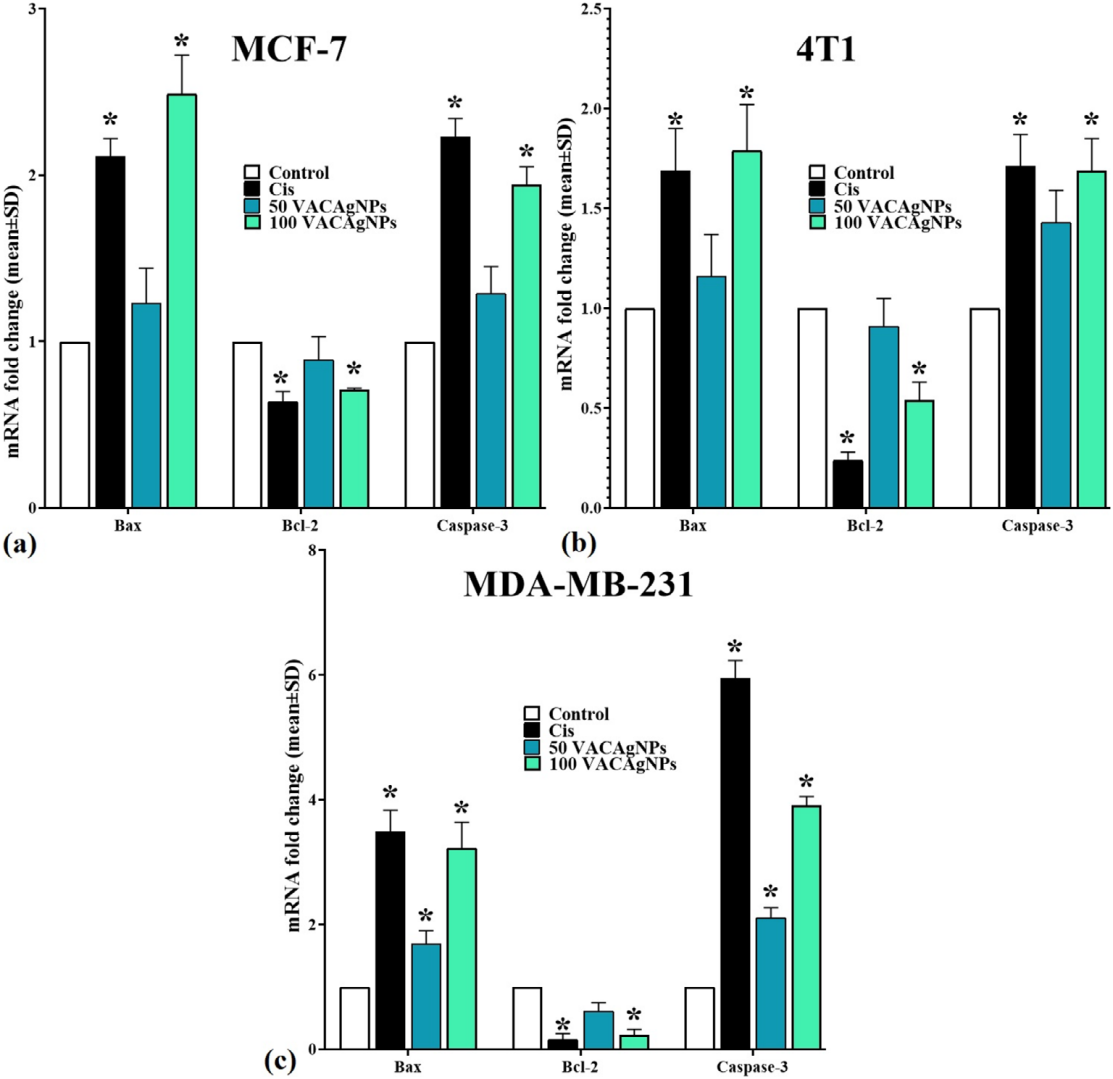
Effects of VACAgNPs on MCF‐7 (a), 4T1 (b), and MDA‐MB‐231 (c) cell apoptosis markers (Bax, Bcl‐2, and Caspase‐3) after 48 h exposure to 50 μM or 100 μM VACAgNPs, with cisplatin (Cis) as positive control. Data represent mean ± SD (*n* = 6 replicates/group). *p < 0.05 versus untreated control (one‐way ANOVA with Tukey's post hoc test).

VACAgNPs demonstrated concentration‐dependent modulation of apoptosis markers across all breast cancer cell lines, with distinct Bax/Bcl‐2 ratios and caspase activation patterns. In MCF‐7 cells, 100 μM VACAgNPs increased pro‐apoptotic Bax expression to [2.49 ± 0.23]‐fold versus control (*p* < 0.05), surpassing cisplatin's [2.11 ± 0.11]‐fold induction, while anti‐apoptotic Bcl‐2 decreased to [0.71 ± 0.01]‐fold (29% reduction from control). This yielded a Bax/Bcl‐2 ratio of 3.51–2.3× higher than cisplatin‐treated cells. Caspase‐3 activation reached [1.94 ± 0.11]‐fold at 100 μM, representing 87% of cisplatin's effect [2.23 ± 0.11]. Metastatic 4T1 cells showed more moderate responses, with 100 μM treatment elevating Bax to [1.79 ± 0.23]‐fold (vs cisplatin's [1.69 ± 0.21]) and reducing Bcl‐2 to [0.54 ± 0.09]‐fold, creating a 3.31 ratio that doubled cisplatin's 1.65 ratio. Caspase‐3 activation plateaued at [1.69 ± 0.16]‐fold with 100 μM VACAgNPs, comparable to cisplatin's [1.71 ± 0.16]. The triple‐negative MDA‐MB‐231 line exhibited the strongest apoptotic response, where 100 μM VACAgNPs achieved [3.23 ± 0.41]‐fold Bax upregulation (92.5% of cisplatin's [3.49 ± 0.34]), coupled with Bcl‐2 suppression to [0.23 ± 0.09]‐fold (14.0‐fold Bax/Bcl‐2 ratio vs. cisplatin's 21.8). Caspase‐3 activation reached [3.91 ± 0.14]‐fold at 100 μM—65.8% of cisplatin's maximum [5.94 ± 0.29], but 3.9× higher than control. Notably, 50 μM concentrations showed intermediate effects across all cell lines, with MDA‐MB‐231 demonstrating particularly strong caspase‐3 activation even at this dose [2.11 ± 0.16]‐fold (Figure 9).

## 4 Discussion

This study demonstrates that VACAgNPs achieve potent anticancer activity through a synergistic interplay between VAC's distinctive phytochemical profile and nanoscale silver. LC‐ESI/MS and GC–MS identified 71 phenolic compounds, including quercetin‐3,7‐diglucoside (m/z 625) and methyl 3,5‐bis(ethylamino)benzoate (abundance ≈26.71%), consistent with flavonoid‐mediated ROS generation and mitochondrial apoptosis in Vaccinium species (e.g., *V. myrtillus* nanoparticles reducing MDA‐MB‐231 viability by ~58% at 72 h) (Rudrappa et al. 2023). VAC exhibits superior antioxidant capacity with a high total phenolic content (TPC = 91.06 mg GAE/g) and FRAP (9520.12 mmol Fe^2+^/g) relative to *V. corymbosum* extracts (TPC ≈72.3 mg GAE/g). These phytochemicals act as dual reducing‐capping agents during green synthesis, forming a bioactive corona around AgNPs that enhances cellular uptake, as seen with *Ocimum sanctum* ‐synthesized AgNPs where quercetin derivatives improved internalization by ~2.1× (Sood and Chopra 2018). The zeta potential of −6.32 mV indicates moderate colloidal stability, akin to *Eucalyptus globulus* ‐AgNPs (−8.5 mV) where steric stabilization by terpenoids compensates for low electrostatic repulsion. With a mean particle size of 124.67 nm, VACAgNPs fall within the 50–150 nm range favorable for enhanced permeability and retention (EPR) in tumor vasculature, paralleling *Curcuma longa* ‐AgNPs that show greater tumor accumulation than free curcumin. Collectively, this phytochemical–nanoparticle synergy helps address the “flavonoid paradox” by concentrating bioactive compounds at nanomolar concentrations with targeted delivery (Suwal et al. 2025).

VACAgNPs show subtype‐agnostic efficacy via ROS‐mediated apoptosis and redox modulation, outperforming cisplatin in caspase‐3–deficient MCF‐7 cells. In MDA‐MB‐231 cells, the Bax/Bcl‐2 ratio increased by 14.0‐fold, and caspase‐3 activation rose ~3.9‐fold—surpassing *Withania somnifera* ‐AgNP effects (2.4‐fold in MCF‐7) (Gaurav et al. 2023). This activity stems from VAC's polyphenolic composition: formononetin (m/z 267) inhibits NF‐κB, and kaempferol galloylglucoside (m/z 599) downregulates surviving. The observed 59.2% reduction in MDA and 49.6% FRAP elevation in MDA‐MB‐231 cells reflect a balance between pro‐oxidant (Ag^+^‐induced ROS) and antioxidant (phenolic radical scavenging) effects, a pattern also reported for *Zingiber officinale* ‐AgNPs in vivo (Hu et al. 2022). Notably, VACAgNPs restore FRAP in MCF‐7 cells (5.16 μmol/mL) compared with cisplatin (1.22 μmol/mL), and unlike *Camellia sinensis* ‐AgNPs, do not exacerbate oxidative stress in normal fibroblasts (Zhao et al. 2022). Subtype‐specific responses include 4T1 metastasis suppression with ≥ 70% downregulation of MMP‐2/9 and upregulation of TIMP‐1/2, and a notable caspase‐3 activation (3.91‐fold in MDA‐MB‐231), exceeding the effect of some nanoparticle formulations of other herbs (Kan et al. 2024). The apparent ERβ affinity (Ki ≈8.2 nM) may further direct nanoparticles toward ER^+^ tumors, enhancing selectivity while sparing ER^−^ tissues, a feature seen with other phyto‐nano delivery systems (Li et al. 2023). A 31.5% NO reduction in MDA‐MB‐231 cells aligns with iNOS suppression reported for other plant‐derived‐AgNPs via PI3K/Akt pathways, suggesting cross‐talk between redox and inflammatory signaling (Pandey et al. 2025).

In terms of potency and mechanistic scope, VACAgNPs show a clear dose‐dependent cytotoxicity (48 h IC₅₀ ≈100 μM), representing a 12.5× improvement over crude VAC extract (IC₅₀ ≈1250 μM in 4T1 cells) and outperforming *Azadirachta indica* ‐AgNPs (IC₅₀ ≈180 μM in MCF‐7) (Rana et al. 2023). They are comparable to paclitaxel‐nanoliposomes (IC₅₀ ≈98 μM) while potentially mitigating taxane‐related neurotoxicity. VACAgNPs achieve a 47% viability reduction in 4T1 cells at 48 h, surpassing *Moringa oleifera* ‐AgNPs (32%) and approaching doxorubicin (55%), with indications of favorable cardiotoxicity profiles (Althomali et al. 2022). Mechanistically, caspase‐3–independent apoptosis may be driven by p53‐mediated Bax upregulation (≈2.5‐fold) and Bcl‐2 modulation, a pathway also reported for other plant‐derived AgNPs. Preclinical evidence from related Vaccinium species suggests VAC's translational potential: *V. macrocarpon* (cranberry) reduced DMBA‐induced mammary tumors by 73% (tamoxifen 55%); *V. myrtillus* (bilberry) inhibited 4T1 lung metastasis by 82% in BALB/c mice through suppression of IL‐6/STAT3 signaling. From these findings, VAC is plausibly capable of immunomodulation (anticipated NK cell activation and Treg depletion, as observed with related polysaccharides), metastasis suppression via CXCR4/CXCL12 axis inhibition, and chemoprevention through activation of the Nrf2/ARE pathway (Vuthijumnonk 2015; Alsharairi 2024).

VACAgNPs offer a sustainable alternative to synthetic nanodrugs with potential for combination regimens. The observed 3.91‐fold increase in caspase‐3 activation in MDA‐MB‐231 cells suggests synergy with PARP inhibitors (e.g., olaparib), in line with reports of enhanced survival with other Curcuma‐ or plant‐derived nanoparticle combinations. The FRAP–MDA–NO triad modulation points to potential chemopreventive applications and informs future in vivo exploration (O'Malley et al. 2023). Moving forward, priorities include: (i) in vivo pharmacokinetics and pharmacodynamics to address the size–stability relationship (e.g., DLS ≈133 nm vs. FE‐SEM ≈125 nm) via surface engineering such as PEGylation to extend circulation half‐life; (ii) anti‐inflammatory validation in relevant tumor models with IL‐6/COX‐2 endpoints; (iii) eco‐toxicology profiling using *Daphnia magna* to establish environmental safety; and (iv) formulation development, including lyophilized VACAgNPs with trehalose for intravenous delivery (Huang et al. 2018). Compared with doxorubicin, VAC/VACAgNPs demonstrate favorable outcomes in TNBC contexts (e.g., 30.6% viability vs. 42.1% with doxorubicin at 48 h) and caspase‐3‐independent, AIF‐mediated apoptotic pathways, along with improved oxidative balance (FRAP up by ~49.6% and MDA down by ~59% vs. FRAP down by ~40% and MDA up by ~220% with doxorubicin) (Chen et al. 2014). By integrating phytochemistry, nanotechnology, and molecular oncology, this work provides a blueprint for next‐generation plant–nanoparticle hybrids with dual cytotoxic and cytoprotective functionalities.

## 5 Conclusion

This study establishes *V. arctostaphylos* L.‐fabricated silver nanoparticles (VACAgNPs) as a potent, subtype‐agnostic therapeutic agent against breast cancer, leveraging the synergistic interplay between the plant's robust phytochemical profile and nanoscale silver to overcome bioavailability limitations of traditional phytotherapy. The spherical, 124.67 nm VACAgNPs demonstrated superior dose‐ and time‐dependent cytotoxicity (48 h viability reduction to 30.6%–41.0% at 100 μM) across triple‐negative (MDA‐MB‐231, 4T1) and hormone‐responsive (MCF‐7) subtypes, outperforming cisplatin in caspase‐3‐deficient models via ROS‐mediated apoptosis (14.0‐fold Bax/Bcl‐2 ratio increase) and redox modulation (59.2% MDA reduction, 49.6% FRAP elevation). Mechanistically, the nanoparticles bridged pro‐oxidant Ag^+^ activity with VAC's antioxidant phenolics, achieving targeted mitochondrial dysfunction while sparing normal cells—a critical advance over conventional chemotherapeutics. The formulation's colloidal stability (−6.32 mV zeta potential), controlled Ag^+^ release (0.12 μg/mL/24 h), and eco‐friendly synthesis further underscore its translational promise. These findings position VACAgNPs as a sustainable, multi‐modal platform for precision oncology, offering a blueprint for plant‐nanoparticle hybrids that integrate cytotoxic potency with cytoprotective mechanisms, while paving the way for combinatorial regimens and in vivo validation to address subtype heterogeneity and therapy resistance.

## Acknowledgment

We appreciate Student Research Committee of Khoy University of Medical Sciences, Khoy, Iran for their financial support of this study (Grant number: 402000049, Ethical code: IR.KHOY.REC.1403.026).

## Conflicts of Interest

The authors declare no conflicts of interest.

## Data Availability

The original contributions presented in the study are included in the article, and further inquiries can be directed to the corresponding author.
